# Correction: Covid-19 in outpatients—Is fever a useful indicator for SARS-CoV-2 infection?

**DOI:** 10.1371/journal.pone.0251623

**Published:** 2021-05-06

**Authors:** Anne Schneider, Holger Kirsten, Franziska Lordick, Florian Lordick, Christoph Lübbert, Amrei von Braun

The following information is missing from the Funding statement: HK was supported by the Federal Ministry of Education and Research Germany (BMBF) within the project PROVID (FKZ 01KI20160C). The funders had no role in study design, data collection and analysis, decision to publish, or preparation of the manuscript.
