# Supplementary figures and images for: Covid-19 in outpatients—Is fever a useful indicator for SARS-CoV-2 infection?
Source: PLoS One. 2021 Feb 3;16(2):e0246312. doi: 10.1371/journal.pone.0246312 (PMC7857607; doi:10.1371/journal.pone.0246312)

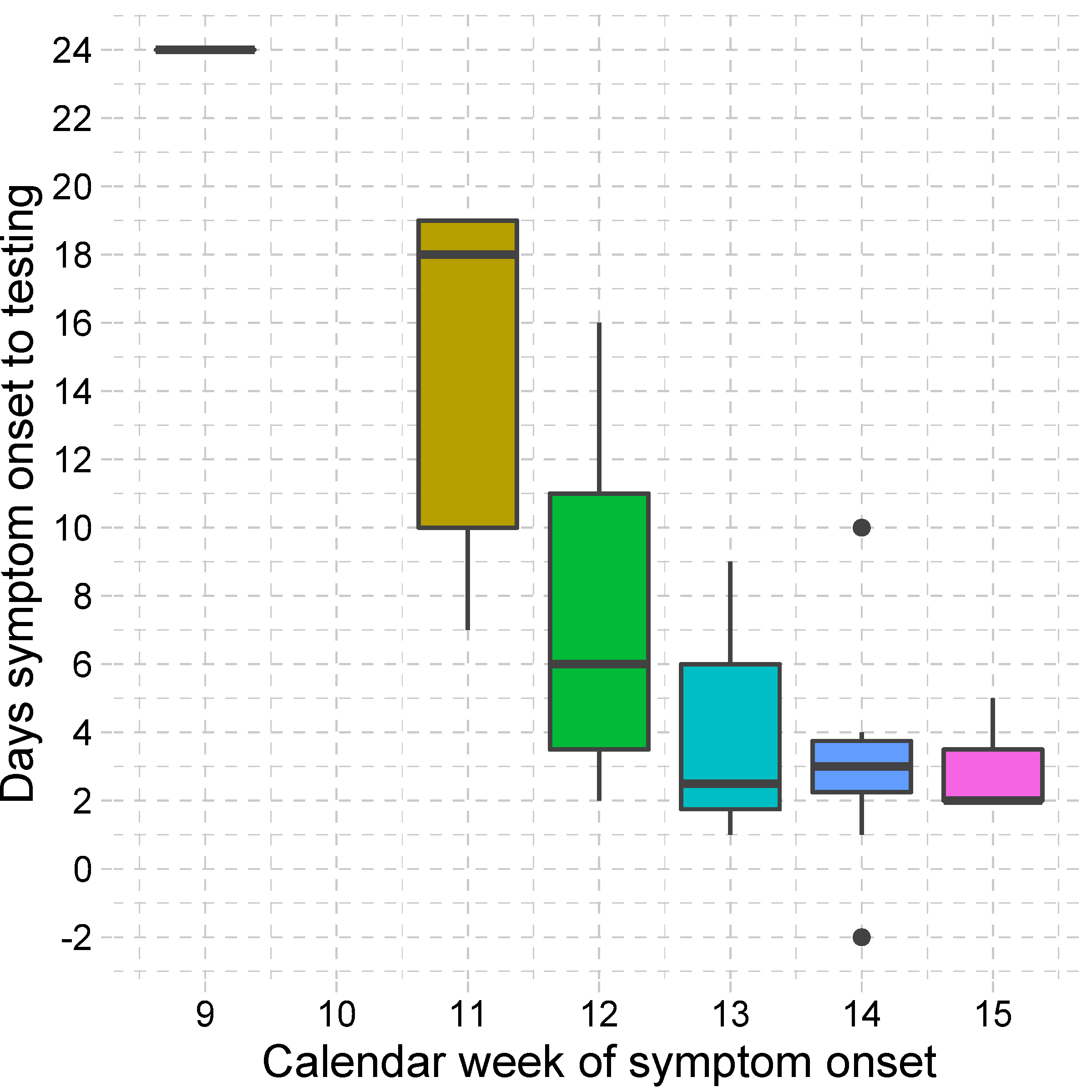

Supplement: S1 Fig — (TIF) [file pone.0246312.s001.tif]
