# Supplementary material for: Covid-19 in outpatients—Is fever a useful indicator for SARS-CoV-2 infection?
Source: PLoS One. 2021 Feb 3;16(2):e0246312. doi: 10.1371/journal.pone.0246312 (PMC7857607; doi:10.1371/journal.pone.0246312)
Supplement: S1 File — (PDF) [file pone.0246312.s002.pdf]

<u>Fragebogen bestätigter COVID-19-Fälle aus der Corona-Ambulanz</u>

<u>Wohnsituation (alleine, Familie, WG etc.)</u>:

<u>Symptombeginn</u>:

<u>Datum des positiven Abstrichs</u>:

<u>Tägliche telefonische Erhebung folgender Symptome bis 14 Tage ab positivem Test</u>:

➔ Bei subj. Verschlechterung Info an Arzt ←

**Datum**							
**Subj. Verschlechterung (j/n)**							
Fieber							
Husten							
Luftnot							
Geruchs-/Geschmacksverlust							
Durchfall							
Allg. Krankheitsgefühl							
Kopfschmerzen							
Andere:							

**Datum**							
**Subj. Verschlechterung (j/n)**							
Fieber							
Husten							
Luftnot							
Geruchs-/Geschmacksverlust							
Durchfall							
Allg. Krankheitsgefühl							
Kopfschmerzen							
Andere:							

<u>Stationäre Aufnahme</u>: *Wann?*: *Wo?*:
