# Supplementary material for: Covid-19 in outpatients—Is fever a useful indicator for SARS-CoV-2 infection?
Source: PLoS One. 2021 Feb 3;16(2):e0246312. doi: 10.1371/journal.pone.0246312 (PMC7857607; doi:10.1371/journal.pone.0246312)
Supplement: S2 File — (PDF) [file pone.0246312.s003.pdf]

<u>Questionnaire</u>

<u>Household members:</u>

<u>Date of first symptoms:</u>

<u>Date of positive swab:</u>

<u>Daily telephone assessment of symptoms for at least 14 days:</u>

➔ In case of progression inform infectious disease doctor ←

**Date**							
**Progression (yes/no)**							
Fever							
Cough							
Dyspnea							
Loss of sense of smell or taste							
Diarrhea							
Feeling unwell							
Headache							
Others							

**Date**							
**Progression (yes/no)**							
Fever							
Cough							
Dyspnea							
Loss of sense of smell or taste							
Diarrhea							
Feeling unwell							
Headache							
Others							

<u>Hospital Admission</u>: *Date* *Where*:
