# Supplementary material for: Covid-19 in outpatients—Is fever a useful indicator for SARS-CoV-2 infection?
Source: PLoS One. 2021 Feb 3;16(2):e0246312. doi: 10.1371/journal.pone.0246312 (PMC7857607; doi:10.1371/journal.pone.0246312)
Supplement: Attachment — Submitted filename: Response to Reviewers.docx [file pone.0246312.s004.docx]

**Response to Reviewers**

*We would like to express our sincere gratitude to the reviewer for their precise review of our manuscript. The comments provided to us are highly appreciated. Kindly find our reply below.*

Reviewer #1: The authors systematically analyzed the symptoms from outpatients with mild to moderate COVID-19 and found that fever was not a common symptom in mild cases. This study is interesting. However, the following major concerns need to be sorted out before it is accepted.

1.The sample size of confirmed covid-19 was small (n=91). Could you add more confirmed mild cases for statistical analysis?

*Response: Thank you for your kind introductory sentence. We completely agree that our sample size is not large enough to make binding statements on clinical screening mechanisms for Covid-19. Larger cohort studies will be necessary to identify such mechanisms based on symptom evaluations in all age groups. However, our study does contribute valuable clinical data on mild to moderate cases which continues to be rare compared to data on severely effected patients.*

*In full agreement with the reviewer, we have added this point to the section on study limitations (page 12, lines 262-266): “(…) while our study contributes to filling the gap in research on Covid-19 in young out-patients, larger cohort studies will be necessary to translate these findings into effective clinical screening mechanisms for Covid-19.”*

*We will unfortunately not be able to include more patients into the analysis as our hospital shifted to in-patient care on the wards and the intensive care units only. Unlike during the first months of the pandemic, out-patient care is now fully covered by general practitioners.*

2.The descriptive bias existed as all these data were obtained by telephone interview. Again, it would be better if the sample size is increased.

*Response: Thank you for pointing this out. Yes, follow-up data was obtained by telephone interview as these patients were subject to official quarantine measures and clinical consultations at our hospital were organized only if necessary. The descriptive bias is reflected in the study limitations (page 12, line 262-263): “Main limitations of our study include the fact that our analysis of symptoms is based on self-reporting only, and that underlying medical conditions were not systematically assessed. “*

*On behalf of all co-authors, I thank you for considering our work in its revised version.*

*Sincerely,*

*Amrei von Braun*
